# Antiviral effect of poly(styrene 4-sulfonate) (PSSNa) on feline calicivirus oral infections in cats—field study

**DOI:** 10.1080/01652176.2026.2616395

**Published:** 2026-01-19

**Authors:** Aleksandra Synowiec, Magdalena Pachota, Martyna Krejmer-Rabalska, Daria Ziemann, Krzysztof Szczubiałka, Michal Jank, Lukasz Rabalski, Maria Nowakowska, Jerzy P. Gawor, Krzysztof Pyrć

**Affiliations:** a Malopolska Centre of Biotechnology, Jagiellonian University, Kraków, Poland; b Acatavir Ltd Company, Kraków, Poland; c Doctoral School of Exact and Natural Sciences, Jagiellonian University, Kraków, Poland; d Laboratory of Recombinant Vaccines, Intercollegiate Faculty of Biotechnology, University of Gdansk, Medical University of Gdansk, Gdansk, Poland; e Veterinary Clinic Arka, Kraków, Poland; f Faculty of Chemistry, Jagiellonian University, Kraków, Poland; g Department of Pre-clinical Sciences and Infectious Diseases, Faculty of Veterinary Medicine and Animal Sciences, Poznań University of Life Sciences, Poznań, Poland; h Biological Threats Identification and Countermeasure Center, General Karol Kaczkowski Military Institute of Hygiene and Epidemiology, Pulawy, Poland

**Keywords:** Feline calicivirus (FCV), poly(sodium 4-styrene sulfonate), PSSNa, field studies, antivirals

## Abstract

Feline calicivirus (FCV) infection causes nasal discharge, oral mucosa inflammation, ulcerations, gingivitis, and conjunctivitis, often progressing to chronic gingivostomatitis, severe pneumonia, and fatal systemic infections. With no antivirals currently available, poly(sodium 4-styrene sulfonate) (PSSNa) was identified in 2019 as a safe inhibitor *in vitro*. In this preliminary single-center, randomized, double-blind, placebo-controlled field study, we further characterized the PSSNa’s safety profile and tested its efficacy in cats after topical oral application. Twenty-eight cats were enrolled in the study, and they were initially treated with standard dental therapy, followed by adjuvant local oral application of PSSNa or placebo. After 4 weeks, PSSNa demonstrated a favorable safety profile with no adverse effects. The treatment group showed a significant decrease in viral load (*p* = 0.001) compared to placebo (*p* = 0.012). Disease symptoms improved significantly, though the oral health index remained unchanged. Additionally, PSSNa showed activity against multiple genetically diverse isolates, indicating a potential, exploratory link between genetic background and treatment outcome. Summarizing, this study presents initial data on the efficacy and tolerability of PSSNa treatment for FCV infections in cats. Nevertheless, several significant limitations should be acknowledged, including inconsistent drug administration by owners, non-sterile housing, sample size, variable oral disease severity, and concurrent treatments.

## GRAPHICAL ABSTRACT

**Figure:**
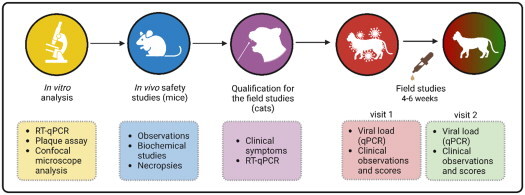

## Introduction

Feline calicivirus (FCV) is a non-enveloped, single-stranded, positive-sense RNA virus classified within the Vesivirus genus of the *Caliciviridae* family. FCV is highly contagious and has become prevalent worldwide in the domestic cat population (Radford et al. 2009). It is considered one of the two leading viral causes of upper-respiratory tract disease (URTD) in cats, which may lead to severe pneumonia (Slaviero et al. 2021). Other symptoms include oral ulceration, hypersalivation, chronic gingivostomatitis (which is considered an immune-mediated condition), conjunctival disorders, and limping syndrome (Dawson et al. 1994; Hofmann-Lehmann et al. 2022; Lanave et al. 2023). Signs usually resolve with time, and symptomatic treatment is employed. However, some FCV strains cause outbreaks with mortality rates reaching up to 60–80% (Schorr-Evans et al. 2003; Hurley et al. 2004; Reynolds et al. 2009; Schulz et al. 2011; Deschamps et al. 2015). Co-infections with feline herpesvirus (FHV-1) are common and have been reported in animals with pneumonia (Monne Rodriguez et al. 2018). Further, FHV-1 infection leads to airway damage and may facilitate secondary infection with FCV due to reduced mucociliary clearance and impaired immune defenses. Interestingly, FCV was also found in feces from cats with enteritis (Di Martino et al. 2020), which might suggest that FCV can acquire gastrointestinal tract tropism and act as an enteric pathogen, similar to other members of the *Caliciviridae* family (Glass et al. 2009; Wilen et al. 2018). There are three open reading frames (ORFs) in the non-segmented FCV genome (Wei et al. 2024; Asif et al. 2025). ORF1 encodes a large polyprotein that is cleaved to generate six non-structural proteins (NSPs), including RNA-dependent RNA polymerase (RdRp), and the genome-linked protein (VPg), essential for viral replication (Sosnovtsev et al. 2002). ORF2 encodes a polyprotein that is subsequently cleaved into the major capsid protein (VP1) and a unique for *Vesivirus* genus capsid leader (LC) protein (Wei et al. 2024; Asif et al. 2025). Finally, ORF3 encodes the minor capsid protein (VP2), which is involved in the virus genome release from the capsid in the cytoplasm of newly infected cells (Conley et al. 2019; Sun et al. 2024).

Antivirals for the treatment of FCV infections are not yet available (Hofmann-Lehmann et al. 2022). Ribavirin was one of the tested candidates; however, despite promising antiviral potency *in vitro* (Li et al. 2020), this compound is toxic to cats (Povey 1978). Several compounds, such as adenosine analog NITD008 (Enosi Tuipulotu et al. 2019), icariin, formononetin, and caffeic acid phenethyl ester (Cui et al. 2021), exhibit high antiviral efficiency against FCV in cell culture; however, no *in vivo* studies are available. Nitazoxanide (NTZ), a drug used to treat parasitic infections in humans, was highly effective against FCV *in vitro* (Fumian et al. 2018; Cui et al. 2020). Moreover, cats experimentally infected with FCV exhibited no adverse effects after the NTZ treatment. The therapy resulted in reduced amounts of the virus in the lungs and trachea and reduced ulcerations in the mouth area, but to the authors’ knowledge, the compound was not yet approved for use in clinic (Cui et al. 2020).

We have previously shown that poly(sodium 4-styrenesulfonate) (PSSNa) is a non-toxic antiviral compound that inhibits the replication of both FCV and FHV-1 *in vitro.* Different mechanisms of action are employed for these two viral species (Synowiec et al. 2019). It interacts with the FHV-1 virion and blocks its interaction with the host’s cell, preventing virus entry. At the same time, the compound interferes mainly with the replication of FCV. As the polymer is highly effective *in vitro*, and we observed no adverse events in our *in vivo* dermal toxicity study in mice, an effort was made to further characterize the efficacy and safety profile of the PSSNa. In this study, 128 cats were preselected with different stages of oral inflammatory diseases, and within this group, 30 were FCV-positive RT-qPCR test (30/128 [23.4%]). Twenty-eight cats met the requirements and were included in the study. These cats were subjected to a single-center, randomized, double-blinded, prospective, and placebo-controlled clinical trial to evaluate the safety and efficacy of the tested product. We observed no adverse effects in the placebo-treated (*n* = 14) nor PSSNa-treated (*n* = 14) group. At the same time, a more significant decrease in viral load and disease symptoms was observed in cats treated for 4 weeks with PSSNa (*p* = 0.001), as compared to the placebo-treated group (*p* = 0.012). Moreover, we demonstrate that PSSNa shows broad specificity against diverse strains of FCV and that there is a correlation between genetic identity and poor treatment outcome. To summarize, PSSNa can be considered a novel supportive antiviral treatment for cats suffering from calicivirus infections.

## Materials and methods

### Cell culture

Crandell Rees Feline Kidney cells (CRFK, ATCC: CCL-94) were maintained in Dulbecco-modified Eagle’s medium (DMEM, high glucose, Life Technologies, Poland) supplemented with 5% heat-inactivated fetal bovine serum (FBS, Life Technologies, Poland) (5% DMEM). Medium was also supplemented with penicillin (100 U/ml, Thermo-Fisher Scientific, Poland) and streptomycin (100 μg/ml, Thermo-Fisher Scientific, Poland) (1% P/S). Cells were cultured at 37 °C in an atmosphere containing 5% CO_2_ and a humidity of 95%. All cells were regularly tested for mycoplasma contamination.

### Viruses

Feline herpesvirus type 1 strain C-27 (FHV-1 C-27; ATCC: VR-636) and feline calicivirus strain F9 (FCV F9; ATCC VR-782™) were used in this study. Virus stocks and mock-infected samples were generated by inoculating fully confluent CRFK cells with a virus or control sample. 24 h (FCV) or 48 h (FHV-1) post-infection (p.i.) supernatants were collected, aliquoted, and stored at −80 °C. Infectious samples were titrated according to the Reed and Muench protocol (Reed and Muench 1938).

### Virus replication assay

In this assay, the antiviral is present before, during, and after the infection, as described previously (Synowiec et al. 2019). CRFK cells were seeded on 96-well plates 24 h before the inoculation. At the moment of the assay, cells were fully confluent. The supernatant was discarded, and 50 μl of fresh medium supplemented with the polymer was added. Plates were incubated for 30 min at 37 °C, and subsequently, the medium with polymer was discarded, and 50 μl of polymer solution or control sample in 5% DMEM with mock or virus (1000 TCID_50_/ml) was added. Plates were incubated for 1.5 h (FCV) or 2 h (FHV-1) at 37 °C, supernatants were then discarded, and cells were washed twice with 1 × PBS (Thermo-Fisher Scientific, Poland). Finally, 100 μl of polymer solution or control sample in 5% DMEM was added to each well, and cells were incubated for 18 h (FCV) or 48 h (FHV-1). After that time, supernatants were collected and subjected to the qPCR analysis.

### Isolation and viral DNA/RNA quantification

A viral DNA/RNA kit (A&A Biotechnology, Gdansk, Poland) was used for viral RNA (or DNA) isolation from swabs or supernatants according to the manufacturer’s instructions. Subsequently, 3 μl of the isolated sample was quantified using 1 × Kapa Probe Fast qPCR MasterMix (Sigma-Aldrich, Poland) for FHV-1 as previously described by us (Synowiec et al. 2019, 2023), or for FCV qPCR coupled with reverse transcription (RT-qPCR) (GoTaq Probe 1-Step RT-qPCR System, Promega, Poland) was used and is detailed below. The reaction mixes for FCV and FHV-1 were prepared according to the manufacturer’s instructions. Specific probe and primers were used to amplify 81 bp sequence fragment of the glycoprotein B (gB) gene of FHV-1 (Vögtlin et al. 2002) or 151 bp region of the FCV genome (nt 5321 to 5471) (Abd-Eldaim et al. 2009). Mix consisted of 200 nM of specific probe labeled with Cyanine-5 (Cy-5) and Black Hole Quencher 2 (BHQ2) (5′-CTT AAA TAY TAT GAT TGG GAY CCC CA-3′), 400 nM of each sense (5′-CAA CCT GCG CTA ACG-3′) and 400 nM of antisense primer (5′-TCC CAY ACA GTT CCA AAT T-3′) for FCV and 200 nM of probe labeled with 5-Carboxyfluorescein (5-FAM) and Black Hole Quencher 1 (BHQ1) (5′-TAT ATG TGT CCA CCA CCT TCA GGA TCT ACT GTC GT-3′), 400 nM of each sense (5′-AGA GGC TAA CGG ACC ATC GA-3′) and 400 nM of antisense primer (5′-GCC CGT GGT GGC TCT AAA C-3′) for FHV-1. The reaction was carried out in a thermocycler (CFX96 Touch™ Real-Time PCR Detection System, Bio-Rad), according to the scheme: 3 min at 95 °C, followed by 39 cycles of 15 s at 95 °C and 30 s at 58 °C (FHV-1) or 51 °C (FCV) as described previously (Synowiec et al. 2019). Eight 10-fold serial dilutions were used as a qPCR template to develop a standard curve as described previously (Pachota et al. 2017; Synowiec et al. 2019). The number of DNA/RNA copies per milliliter was calculated using the approximate molecular weight of deoxyribonucleotide (320 g/mol) and Avogadro’s constant.

### cDNA synthesis, PCR amplification, and library preparation

Viral RNA from swabs (FCV-positive cats) was subjected to reverse transcription using Luna Script RT SuperMix (New England BioLabs, USA) according to the manufacturer’s protocol. The resulting cDNA was used as a template for downstream amplification reactions. cDNA was used for amplification of fragments of ORF1 and ORF2 from the FCV genomes in PCR reactions with a set of internally developed primers. PCR products for each sample of ∼1000–2000 bp in length were purified using magnetic beads. DNA was barcoded with the Rapid Barcoding Kit SQK-RBK114-96 and sequenced on a MinION Mk1B equipped with a FLO-MIN114 (R10.4.1) flow cell (run duration 96 h, ‘reserved pores’ enabled, 1.5 h pore scans) under MinKNOW v24.11.10 (https://nanoporetech.com/document/experiment-companion-minknow).

### Sequencing and data processing

High-accuracy live basecalling was performed by Dorado v7.6.8 (https://github.com/nanoporetech/dorado) using model r1041_e82_400bps_sup_v4.3.0 with a minimum Q-score of 10. The Dorado reads were then processed with BBDuk v38.96 (Bushnell, 2014, https://sourceforge.net/projects/bbmap/) to remove primer sequences from read ends and Filtlong v0.2.1 (https://github.com/rrwick/Filtlong) to remove low-quality reads and those shorter than 200 bp.

### Genome assembly and polishing

This filtered dataset was assembled *de novo* with Flye v2.9.5 (Kolmogorov et al. 2020) and polished with Medaka v2.0.0 (https://github.com/nanoporetech/medaka) using the matching model. Subsequently, reference sequences from NCBI were aligned to these assemblies, and sequences were reassembled based on individual references using Minimap2 v2.28 (Li 2018) and Medaka. The resulting sequences served as input for phylogenetic analysis. Nucleotide sequences were translated to amino acid sequences, with ambiguous residues represented as ‘X’ characters where sequencing quality was insufficient. Sequences containing >50% missing data in specific regions were excluded from downstream analyses to ensure data quality.

### Sequence alignment and phylogenetic analysis

Multiple sequence alignments were performed using MAFFT v7.526 (Katoh and Standley 2013) with auto-detection of alignment strategy to optimize accuracy for the given sequence characteristics. Maximum likelihood phylogenetic reconstruction was performed using IQ-TREE v3.0.1 (Wong et al. 2025) with automatic model selection based on the Bayesian Information Criterion (BIC). The analysis included >200 available FCV sequences from public databases (NCBI GenBank) to provide comprehensive evolutionary context. Branch support was assessed using two complementary methods: 1000 ultrafast bootstrap replicates and SH-like approximate likelihood ratio test (Guindon et al. 2010).

Separate phylogenetic analyses were conducted for ORF1 and ORF2 regions to assess whether drug resistance phenotypes correspond to viral genetic relationships and to identify which genomic regions may harbor resistance determinants. Trees were rooted using reference strain NC_075569 and visualized with branch lengths proportional to evolutionary distance measured in substitutions per site.

### Compound preparation

Poly(sodium 4-styrenesulfonate) (PSSNa) with a weight average of ∼1,000,000 Da was purchased from Merck (Poland). The PSSNa formulations were prepared by Pikralida Ltd (Poznan, Poland) as described below. Formulation No. 1: PSSNa was diluted to a concentration of 30 mg/ml in water and gelling substance, 3% (w/v) carmellose sodium (Ashland). Citric acid (Chempur) was added to adjust the pH to 7.4. Finally, glycerin (Biomus) was added as a moisturizing substance (final concentration 5% (w/v)). Formulation No. 2: PSSNa was diluted to a concentration of 30 mg/ml in water and gelling substance, 1.5% (w/v) Carbopol^®^ 974 P NF Polymer (Lubrizol). Triethylamine (TEA, Chempur) was added to adjust the pH to 7.4. Finally, glycerin (Biomus) was added as a moisturizing substance (final concentration 5% (w/v)). Formulation No. 3: First, a moisturizing substance, glycerin (Biomus), was mixed with dH_2_O. Then, PSSNa was added gradually until the active compound reached the final concentration and was completely dissolved. Subsequently, Hypromellose 2208 was used as a gelling substance and was progressively added until the final concentration was reached and the dissolving of the substance was complete. The final concentrations were as follows: PSSNa 30 mg/ml, 5% (w/v) glycerin (Biomus), and 1% (w/v) hypromellose 2208 (Ashland). Finally, citric acid (Chempur) was added to adjust the pH to 7.4.

The placebo was prepared in the same manner as relevant formulation, except for adding PSSNa. Additionally, gamma irradiation (dose of 10 Gy) was used as a method of sterilization for both PSSNa and placebo formulations, which were then placed in sterile 10 ml syringes. Syringes were stored at +4 °C until further use. Gamma irradiation was performed at the Institute of Nuclear Chemistry and Technology in Warsaw, Poland. After the sterilization, PSSNa and placebo were subjected to (1) microbiological purity tests, including the determination of all parameters specified in the international standard PN-EN ISO 17516:2014; (2) the aging and packaging compliance tests; (3) human skin irritation tests. The tests were performed by an external company (Ekolabos Ltd, Wroclaw, Poland).

### Aging and packaging compliance test

The research was conducted in compliance with the legal framework established by Regulation (EC) No 1223/2009 of the European Parliament and of the Council of 30 November 2009 on cosmetic products, as well as the Act of October 4, 2018, on cosmetic products (Journal of Laws 2018, item 2227). The study also adhered to the guidelines provided by the European Cosmetic Toiletry and Perfumery Association (COLIPA) in their *Guidelines on Stability Testing of Cosmetic Products* (March 2004). The testing procedure followed PBW 46, edition 1, dated September 11, 2015, which involves evaluating the stability and compatibility of the cosmetic product mass with its packaging. Stability tests were conducted using accelerated aging methods by exposing the product to an elevated temperature of 45 ± 1 °C for a period of 8 weeks. The duration of the tests was determined based on the estimated shelf life provided by the Responsible Entity and considering van ‘t Hoff’s rule, which states that the rate of chemical reactions increases 2 to 4 times for every 10 °C rise in temperature. The product was also placed in alternative packaging to compare the seal integrity of the original and substitute packaging and to verify the absence of any reactions between the cosmetic product and the packaging material. Control samples, stored at room temperature in a dark location, were included in the study for comparison.

### Human skin irritation test

The study was conducted in accordance with Regulation (EC) No. 1223/2009 of the European Parliament and of the Council on Cosmetic Products, as well as the guidelines provided by Cosmetics Europe—The Personal Care Association (*Product Test Guidelines for the Assessment of Human Skin Compatibility, 1997*). Ethical principles outlined in the WMA Declaration of Helsinki (editions 1964–2013) were strictly followed. Additionally, the research adhered to the Act on Cosmetic Products of October 4, 2018 (Dz.U. 2018, item 2227), along with the internal procedures and technical instructions of SKINLAB P.S.A., including PO-08 (*Realization of Research Studies*), I02/PO-08 (*Dermatological Testing—Patch Tests*), and I04/PO-08 (*Evaluation Scheme for Skin Reactions—Product Classification*). Volunteer participants were selected in compliance with current Polish and European legal regulations, the guidelines of Cosmetics Europe—The Personal Care Association, and the principles of the WMA Declaration of Helsinki (editions 1964–2013). The selection process followed the research procedure PO-08 (*Realization of Research Studies*) and the technical instruction I01/PO-08 (*Qualification of Volunteer Subjects for the Study*) as established by SKINLAB P.S.A. All volunteers met the inclusion criteria for participation, provided written informed consent, and were fully informed about the study’s purpose, methodology, and potential adverse effects. Throughout the entire study, the volunteers were under the constant supervision of a dermatologist. The study utilized a Jadassohn-Bloch skin test model modified by Rudzki. This method involved a single application of the test product to a designated skin area, followed by periodic observations to evaluate the skin’s condition. Results were recorded, and the product was classified based on a point scale (0–4) for skin reactions, as specified in the I04/PO-08 classification system. The qualifications of volunteers, application of the test samples, and evaluation of results were conducted at SKINLAB P.S.A. in Kraków. Each study group consisted of 25 volunteers.

### Toxicity studies in mice

Mice were handled under good animal practices defined by the relevant national and local animal welfare bodies. All mice work was approved by the Local Ethical Committee for Animal Research (approval 68/2021) and was performed in the Animal Facility of Malopolska Centre of Biotechnology, Jagiellonian University, Krakow, Poland. Toxicity studies were conducted on 6-week-old female BALB/c mice (The Center for Experimental Medicine of the Medical University of Bialystok). The animals were housed individually at 22 ± 2 °C with a relative humidity of 55 ± 5% and 20 air changes per hour. The animal room was maintained on 12 h light and dark photoperiodic cycles.

The PSSNa formulation was prepared as described in the previous section. The toxicity studies were conducted using PSSNa formulation containing 0 (placebo), 5, 15, or 30 mg/ml of active compound, the starting dose was the same as in previous *in vivo* studies (Herold et al. 2000). After 5 days of quarantine, mice were clustered into eight experimental groups (*n* = 5) and were treated with different polymer concentrations (0 (placebo control), 5, 15, and 30 mg/ml). Two routes of administration were tested: intragastric and on-the-eye. The experiment lasted 14 days, and the formulation was administered daily. Mice were monitored and weighed daily. After 14 days from the first application, animals were euthanized by cervical dislocation. The blood, liver, kidney, and spleen were collected from euthanized mice for further analysis. The biochemical analysis, including GLU, BUN, ALP, TP, GPT, and CRE, was performed on days 7 and 14. The study was carried out in two independent replications.

### Case selection for field study

Case selection was performed at ARKA Veterinary Clinic in Cracow between February 2022 and February 2023. Client-owned cats with the following inclusion criteria were selected for the study: presence of feline chronic gingivitis and FCV-positive RT-qPCR tests. As the study was performed according to the best standards of care, the Local Ethical Committee for Animal Research waived a requirement for its approval. Each cat’s owner provided written consent before a cat was included in the study.

### Clinical study design

All cats included in this study first received oral treatment according to the best standards of veterinary dental care and performed by board certified veterinary dentistry specialist (JG) and resident of the European Veterinary Dental College (DZ). The owner compliance form was established and used throughout the study. It included a declaration of daily administration of the product, explanation in case of missing its administration, a column dedicated to any side effects observed, and any other remarks from the owner about the animal’s status. Every owner was urged to present a treated animal in case of health problems like anorexia, oral pain, or issues associated with the alimentary tract. All the owners were instructed on how to apply the gel to the cat’s oral cavity, which was administered once daily in an amount of 2 drops. Parameters were recorded and scored, utilizing the assessment of the Disease Improvement Score (DIS, including GBI - Gingival Bleeding Index, ulceration surface, and periodontitis) and the Oral Health Index (OHI, including the size of mandibular lymph nodes on palpation, the presence of dental deposits, and the presence of periodontal disease). The parameters are summarized in Table 1.

**Table 1.** Oral health parameters were assessed during patient examination in this study.

Category		Score		
		0	1	2
Disease improvement score (DIS)	Gingival Bleeding Index (GBI)* (Lobene et al. 1986)	>60% of an improvement (compared to Day 0)	60–30% of an improvement (compared to Day 0)	<30% of an improvement or no improvement (compared to Day 0)
	Ulceration	>60% of an improvement (compared to Day 0)	60–30% of an improvement (compared to Day 0)	<30% of an improvement or no improvement (compared to Day 0)
	Intensity of inflammation	>60% of an improvement (compared to Day 0)	60–30% of an improvement (compared to Day 0)	<30% of an improvement or no improvement (compared to Day 0)
Disease score (Oral Health Index, OHI^#^) (Gawor et al. 2006)	Size of mandibular lymph nodes on palpitation	Normal	Slightly enlarged	Moderately to severely enlarged
	Presence of dental deposits (plaque, calculus)*	Absent	≤50% of dental crowns affected	>50% of the dental crowns affected
	Presence of periodontal disease	Absent	Gingivitis	Periodontitis

*For cats that did not receive dental extractions.

^#^The sum of scores obtained for the three parameters provide OHI: 0 points indicate optimal oral health, and 6 points indicate the poorest oral health (Gawor et al. 2006).

### Swabs collection and processing

Swabs from the oropharynx were collected from cats and placed into the sterile swab tubes (clean swab tube 12 × 133 mm with aluminum rod and viscose swab in transport tube, Meus, Poland) at the time of qualification and 4 weeks after receiving treatment. Swabs were immediately transported to the laboratory or stored at −20 °C. 1 ml of 1 × PBS (Thermo Fisher Scientific, Poland) was added to the vial tube, and the swab was vortexed and incubated for 10 min at RT. Subsequently, 100 µl of elution was used for viral RNA isolation, and the rest was stored at −80 °C.

### Statistical analysis

Statistical calculations were performed using the GraphPad Prism 9 software. Data are presented as the mean ± *SD* (standard deviation) or mean SEM (standard error of the mean) for parametric datasets and median ± IQR (interquartile range) for non-parametric datasets. The normality of data was evaluated using the Shapiro-Wilk test, and the *F*-test was used to test the assumption of equality of variances. A value of *p* < 0.05 was considered significant for all analyses.

## Results

### PSSNa activity is retained in co-infections with FCV and FHV-1

**Figure 1.**
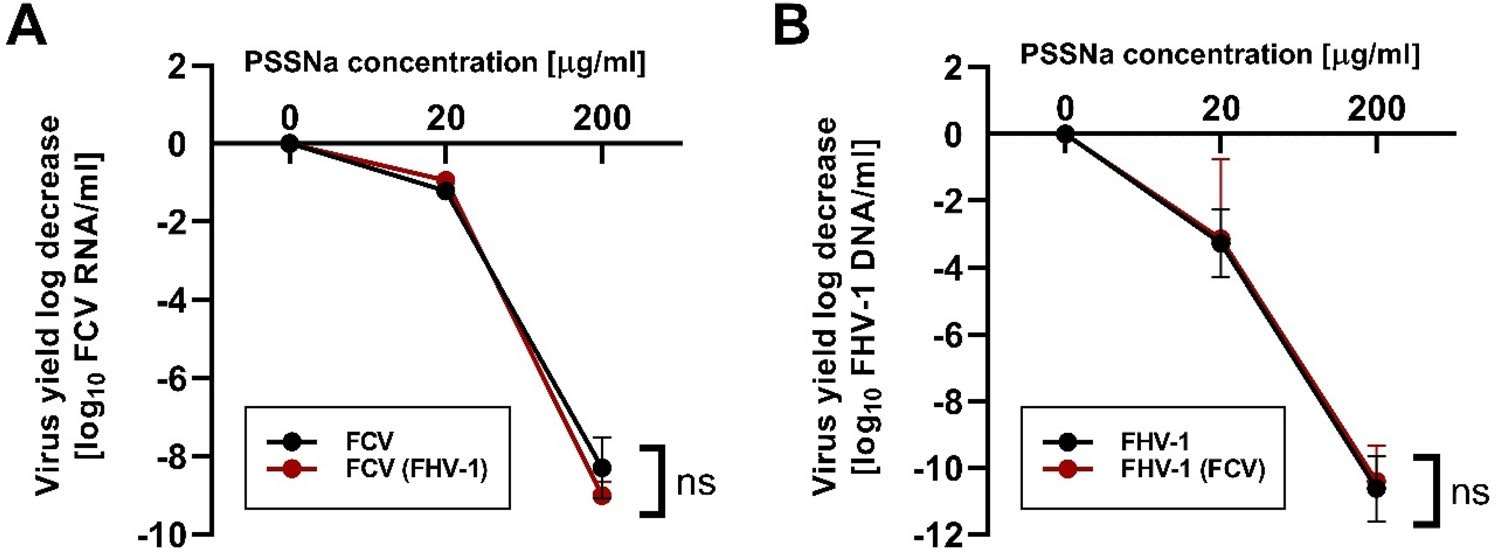
PSSNa activity is retained during co-infections with both FCV and FHV-1. Experiments were carried out at a concentration of 200 or 20 μg/ml for time points: (A) 24 h (for FCV) and (B) 48 h (for FHV-1). Virus yield was assessed by RT-qPCR or qPCR, and the data are presented as a log_10_ change of virus RNA/DNA copies/ml. Results were normalized to the values obtained for untreated, infected cells and are presented as mean ± SEM. The data presented were collected in three independent experiments, each performed at least in duplicate. The two-way ANOVA test was applied to determine the significance of differences between groups. ns: not significant (*p* > 0.05).

We have shown previously that PSSNa is highly effective for treating both FCV and FHV-1 infections *in vitro* (Synowiec et al. 2019). However, co-infections with FCV and FHV-1 are common in cats (Henzel et al. 2012; Monne Rodriguez et al. 2018). To complement our previous study, we performed experiments evaluating the efficacy of the polymer during co-infection *in vitro*. We infected CRFK cells at 1,000 TCID_50_/ml with FCV, FHV-1, or FCV and FHV-1 together in the presence of polymer at 0, 20, or 200 µg/ml concentrations. As the FCV-related cytopathic effect (CPE) occurs faster, samples were collected at two different time points, 24 h (see Figure 1(A)) and 48 h (see Figure 1(B)). We observed no significant difference in the efficacy of PSSNa between single infections and co-infections.

### PSSNa is safe when administered intragastrically and on the eye in mice and also does not cause any dermal irritation in humans

We previously observed no dermal toxicity of the polymer in the mice model (Synowiec et al. 2019). Nevertheless, for the field trials in target species (cats), it was essential to assess the intragastric and eye toxicity as these are the main areas affected by feline URTD, apart from the skin. Additionally, we prepared three different formulations to select the optimal one for cats. The antiviral activity of all three formulations was assessed by the analysis of CPE development in CRFK cells. The PSSNa retained its antiviral activity in all formulations (see Supplementary Figures 1(A,B)). Based on the formulation’s viscosity optimal for administration in animals, we chose formulation no. 3 (as per veterinarians’ assessment). Before further study, formulation no. 3 and a corresponding placebo were subjected to sterilization by gamma irradiation. The microbiological purity tests showed that formulation no. 3 and placebo after the sterilization were free from microbiological contaminations (see Supplementary Material 1). Additionally, we subjected the sterile product to the aging and package compliance test. This test showed that the product is stable and compatible with the packaging (see Supplementary Material 2). The final product’s safety profile was evaluated in the BALB/C mice model. The PSSNa or placebo was administered daily for 14 days, and mice were monitored daily. No adverse effects were recorded at 0 (placebo), 5, 15, or 30 mg/ml, and no alterations in behavior or appearance were observed throughout the experiment. Animals’ anesthesia was performed on day 14, and the necropsy did not reveal any alteration of the internal organs (eye, stomach, liver, thymus, kidneys, adrenal glands, intestines, and lungs). We did not observe any significant body mass change (see Supplementary Figures 2(A,B)) or abnormalities in biochemical blood analysis (see Supplementary Figures 3(A–L)). Additionally, since cat owners may come into exposure to the compound through skin-to-cat fur contact, we decided to check whether an allergic reaction in humans through skin contact could occur. We included 100 participants, randomly assigned into four groups, 25 participants each. Half of the participants had normal skin type, and the other half had sensitive skin type. Neither PSSNa nor placebo exhibited any allergic or skin-irritating effects in any tested human participants, regardless of skin type (see Supplementary Material 3).

### PSSNa is safe and significantly decreases viral load in cats

To evaluate the antiviral activity of PSSNa in cats, we carried out a randomized, double-blinded, prospective, and placebo-controlled field study. First, 128 cats with clinical signalments of chronic or acute periodontitis were tested for the presence of FCV and FHV-1 viruses using RT-qPCR. In this population, 30 cats tested positive for FCV (23%), and finally, 28 of them (22%) were qualified for and completed the field study with all the required visits. Of note, only four cats (3%) tested positive for FHV-1; however, this group was too small for this field study to be statistically relevant. PSSNa-treated and placebo-treated groups comprised 14 cats each, and the animals were randomly assigned to groups. Between the two groups, there were no significant differences either in age (*p* = 0.10, two-tailed *t*-test), sex (*p* = 0.70, two-sided chi-square test), or body weight (*p* = 0.77, two-tailed *t*-test). The descriptive data for cats is summarized in Table 2. No adverse effects were observed in any cat treated with PSSNa or placebo throughout the study.

**Table 2.** Descriptive data for cats with chronic gingivostomatitis enrolled in a randomized, placebo-controlled clinical trial of PSSNa.

Parameter		Group	
		PSSNa (*n* = 14)	Placebo (*n* = 14)
Sex	Male	7 (50%)	8 (57%)
	Female	7 (50%)	6 (43%)
Age in years	Median (range)	3.5 (0.5–7.5)	4.75 (1.0–12.0)
Weight in kg	Median (range)	3.98 (2.88–7.49)	4.35 (2.82–5.70)
Primary therapy during the study	FMX	4 (29%)	2 (14%)
	PMX	7 (50%)	8 (57%)
	MT	3 (21%)	2 (14%)
	RAD + PRO	0	2 (14%)

FMX: full mouth extraction; PMX: partial mouth extraction; MT: medical treatment; RAD: radiographic assessment; PRO: professional dental cleaning.

**Figure 2.**
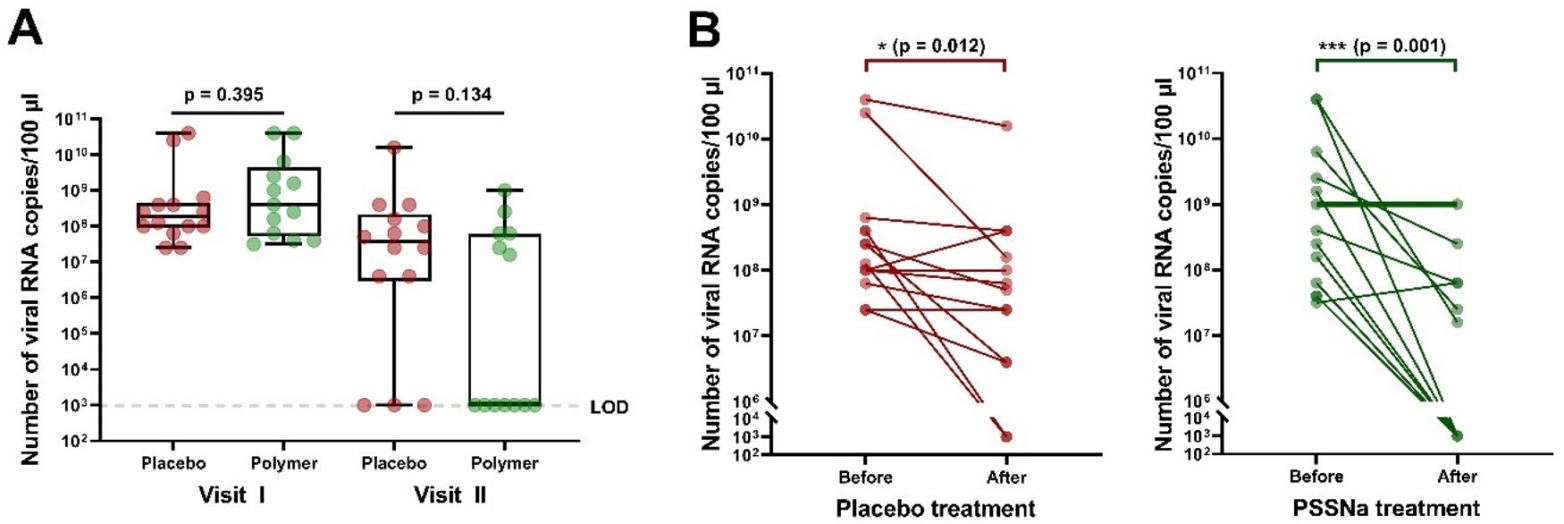
Quantitative evaluation of antiviral effects of PSSNa in cats. Cats were administered PSSNa or placebo daily for 4 weeks. Before and after the treatment, cats were swabbed, and viral load was evaluated by RT-qPCR. (A) The viral load is presented as a number of viral RNA copies/100 µl. The box plot shows the median (line within the box), interquartile range (box borders), and min/max values (whiskers). (B) Each cat’s viral load change is presented, and a bolded line indicates the cat (case 28) from which the virus was isolated. Group differences were compared using a paired, two-tailed, non-parametric Wilcoxon test. Values statistically significant (*p < 0.05 or ***p < 0.001) are indicated by asterisks. LOD: limit of detection.

The swabs were taken from every cat included in the study before the drug administration and after 4 weeks of treatment. Swabs were analyzed by qPCR, and the results are presented in Figures 2(A,B). All the cats included in the studies received primary therapy, as depicted in Table 2. Every cat received a comprehensive oral health assessment, both clinical and radiographic. Based on these assessments further decisions were made and definite treatments elected: full mouth extraction (FMX) which was extraction of all teeth; partial mouth extraction (PMX) which was extraction only of teeth affected by periodontal attachment loss; medical treatment (MT) which included anti-inflammatory drugs, application of recombined interferon omega (Virbagen Virbac), or using immunostimulatory drug (Zylexis Zoetis) (Hennet et al. 2011; Jennings et al. 2015; Anderson and Hennet 2022; Soltero-Rivera et al. 2023). All cats after oral surgery procedures (FMX, PMX), as well as those with signs of oral pain, received pain management protocol according to the best standards of care (Steagall et al. 2022; Monteiro et al. 2023). At the top of primary therapy, every cat received topical use of either placebo or PSSNa. The primary treatment was limited to professional dental cleaning and radiographic assessment received by 2 cats, both from the placebo group (*n* = 2). Only medical treatment received 2 cats from the placebo group and 3 from the treated group (*n* = 5). PMX was performed in 7 cats from the placebo group and in 8 from the PSSNa treated group (*n* = 15). All teeth were extracted in 4 cats from the treated group and 2 from the placebo group (*n* = 6). No significant differences in viral load were observed for the groups at the beginning of the study (*p* = 0.66, unpaired *t*-test). On top of the primary treatment, cats were administered with PSSNa or a placebo as an adjunct therapy. As expected, the viral load was decreased in both groups due to natural healing processes in some animals; however, we observed a greater decrease in viral loads in PSSNa-treated cats (***p = 0.001) than in placebo-treated cats (*p = 0.012). Moreover, in the PSSNa-treated group, seven cats (7/14, 50%) were FCV-free after the treatment, compared to only three cats (3/14, 22%) in the placebo-treated group. The animals were divided into three groups subjected to each based on the thorough clinical oral assessment: A—gingivitis individuals (*N* = 9; 5 PSSNa-treated and 4 placebo-treated); B—gingivostomatitis (cats with inflammatory disease and having teeth; *N* = 17; 7 PSSNa-treated and 10 placebo-treated); C—caudal stomatitis (cats without teeth but with inflammatory disease; *N* = 2). In group A, we observed a decrease in viral load in 80% of animals (4/5 cats) treated with PSSNa (notably, 60% (3/5 cats) were FCV-free). Additionally, one cat (1/5) treated with PSSNa was reported to have a slight increase in viral load. In the placebo-treated population, within group A, in 25% (1/4) of cats no change; in 50% (2/4 cats) of cats only a slight decrease; and in 25% (1/4) of cats, a significant decrease was recorded. Within group B, all (6/6, one cat’s swab could not be processed due to the technical error) PSSNa-treated cats exhibited a reduced viral load, compared to 60% (6/10) of placebo-treated cats. Additionally, 50% of animals (3/6 cats) in the PSSNa-treated group were virus-free, and a vast decrease was recorded in 33% (a further two cats). In contrast, in the placebo-treated group, only 20% (2/10 cats) were virus-free, and a vast decrease in virus load was noted for a further 20% (2/10 cats). No change in viral load was reported in 40% of animals (4/10 cats).

Interestingly, the antiviral effect was not observed in every PSSNa-treated cat. We decided to isolate one FCV strain from a PSSNa-treated cat that did not show improvement in viral load throughout the study (case 28), despite teeth extraction and a slight improvement in OHI. Next, we compared the antiviral effect of PSSNa on laboratory strain F9 and one isolated FCV clinical strain from a cat with resistant infection (bolded line in Figure 2(B)). As expected, we observed complete inhibition of FCV F9 replication; however, we did not observe the same phenomenon for the clinical strain (see Supplementary Figure 4). Clinical strain replication was inhibited by ∼85% at the concentration of 64 µg/ml, and no further dose-dependency was recorded despite increasing PSSNa concentration. This phenomenon was not observed in another clinical strain we tested in our previous study (Synowiec et al. 2019).

### PSSNa treatment improves disease-specific clinical signalments but does not improve the general Oral Health Index (OHI)

**Figure 3.**
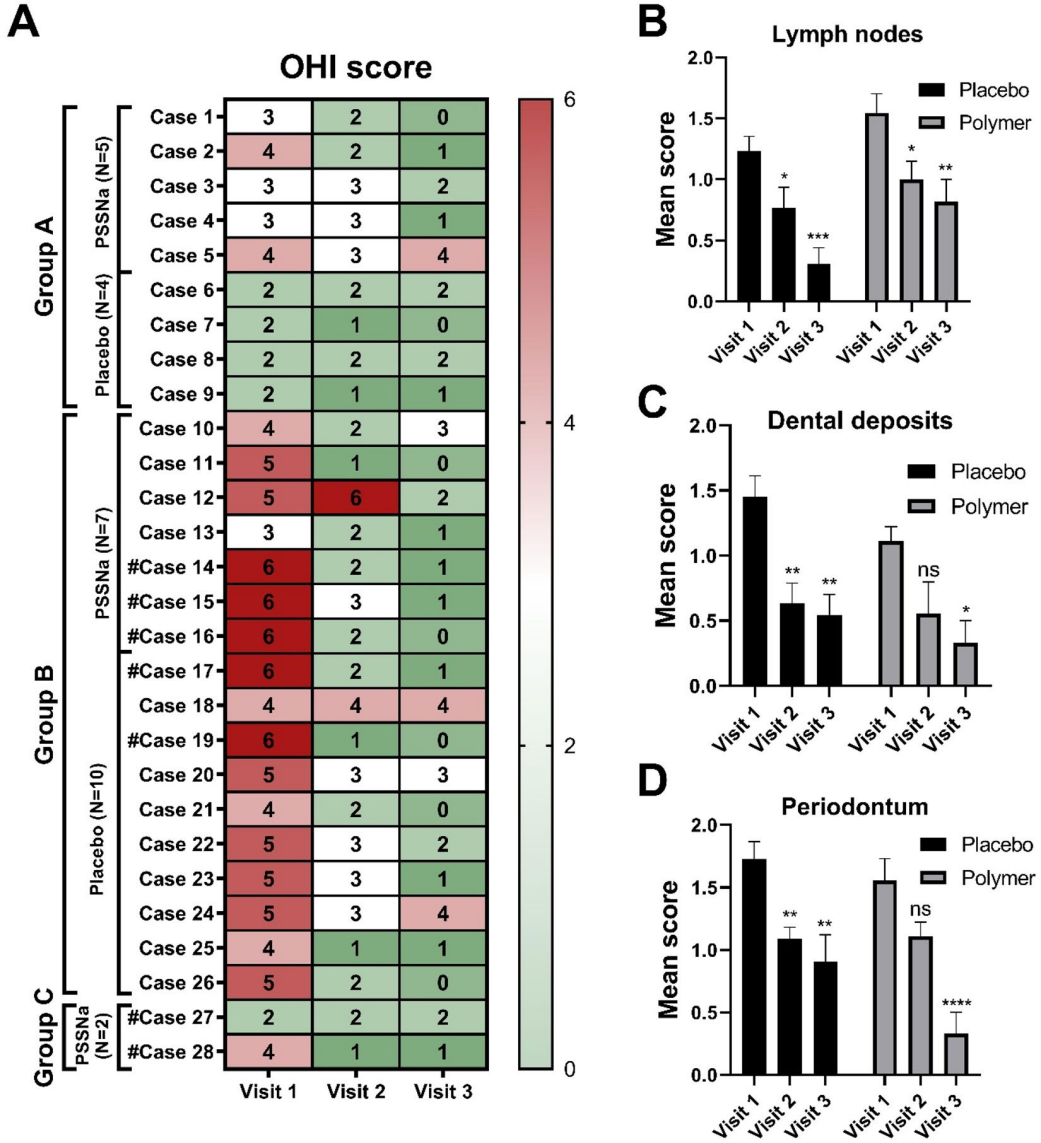
Changes in oral health index (OHI) (Gawor et al. 2006). (A) OHI [lymph nodes mean score (0–2) + dental deposits mean score (0–2) + periodontium mean score (0–2)] between visit 1 (week 0) and visit 2 (week 2) or visit 3 (week 4) are presented for each case separately in the form of a heat map for all groups (group A: juvenile gingivitis or older individuals; group B: gingivostomatitis, cats with teeth; group C: caudal stomatitis). ^#^The periodontium and dental deposit scores were not included for cases 14, 15, 16, 17, 19, 27, and 28, as these cats had their teeth extracted. Lymph nodes (B), dental deposits (C), and periodontium (D) mean improvement scores are presented for both placebo-treated (*N* = 14; *N* = 12 for dental deposits and periodontium) and polymer-treated (*N* = 14; *N* = 9 for dental deposits and periodontium) groups of cats. 2—small improvement/no improvement, 1—medium improvement, and 0—significant improvement/complete recovery. Data are presented as mean ± SEM. Differences between groups were compared using the RM two-way ANOVA with Bonferroni’s multiple comparisons test. ns: not significant (*p* > 0.05), *p < 0.05, **p < 0.01, ***p < 0.001, ****p < 0.0001.

**Figure 4.**
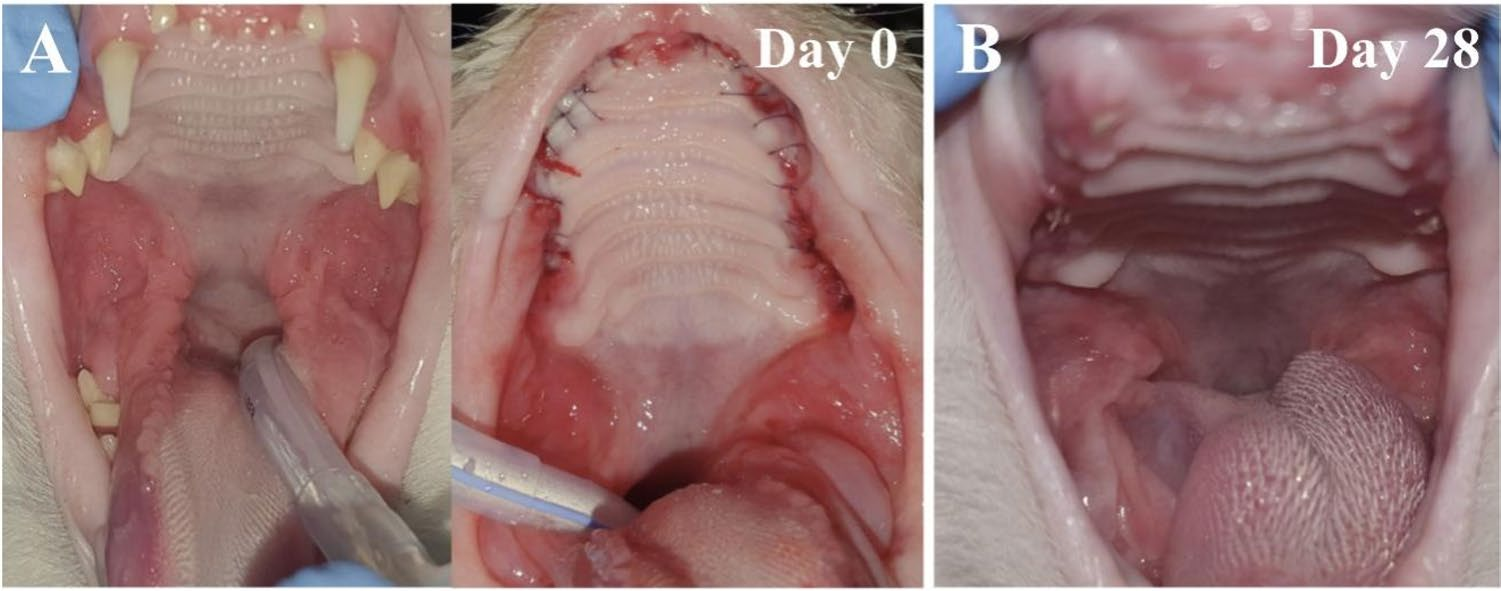
Cat FCV-positive from the placebo-treated group (case 17, group B). (A) Day 0 features gingivitis and caudal mucositis with ulcerations (left panel). Postoperative image after performing full mouth extraction (FMX) (right panel); (B) and 28 days after application of placebo. Improved clinical status but ulcerations and inflamed area still visible.

The Oral Health Index (OHI) takes into account the lymph node condition, dental deposits, and periodontium. This score was assessed and calculated separately on visit 1, visit 2, and visit 3. We observed a general improvement in OHI of all animals, regardless of the treatment, but no differences between placebo-treated and polymer-treated groups. Complete recovery was observed in 3/14 (21%) PSSNa-treated cats and in 4/14 (29%) of placebo-treated cats. The individual changes in OHI scores of each case are presented in Figure 3(A). The mean improvement scores for the condition of lymph nodes, dental deposits, and periodontium are presented in Figures 3(B–D), respectively. The periodontium and dental deposit scores were not included for cases 14, 15, 16, 17, 19, 27, and 28, as these cats had their teeth extracted. The representative pictures of the oral cavity of the placebo-treated cat (case 17) are presented in Figure 4. The general OHI improvement is noticeable. On day 0, the cat features gingivitis and caudal mucositis with ulcerations (see Figure 4(A), left panel). The postoperative image taken after performing FMX is shown in Figure 4(A), right panel, and 28 days after the application of the placebo, the improvement in clinical status is visible. However, ulcerations and inflamed areas are still present (see Figure 4(B)).

**Figure 5.**
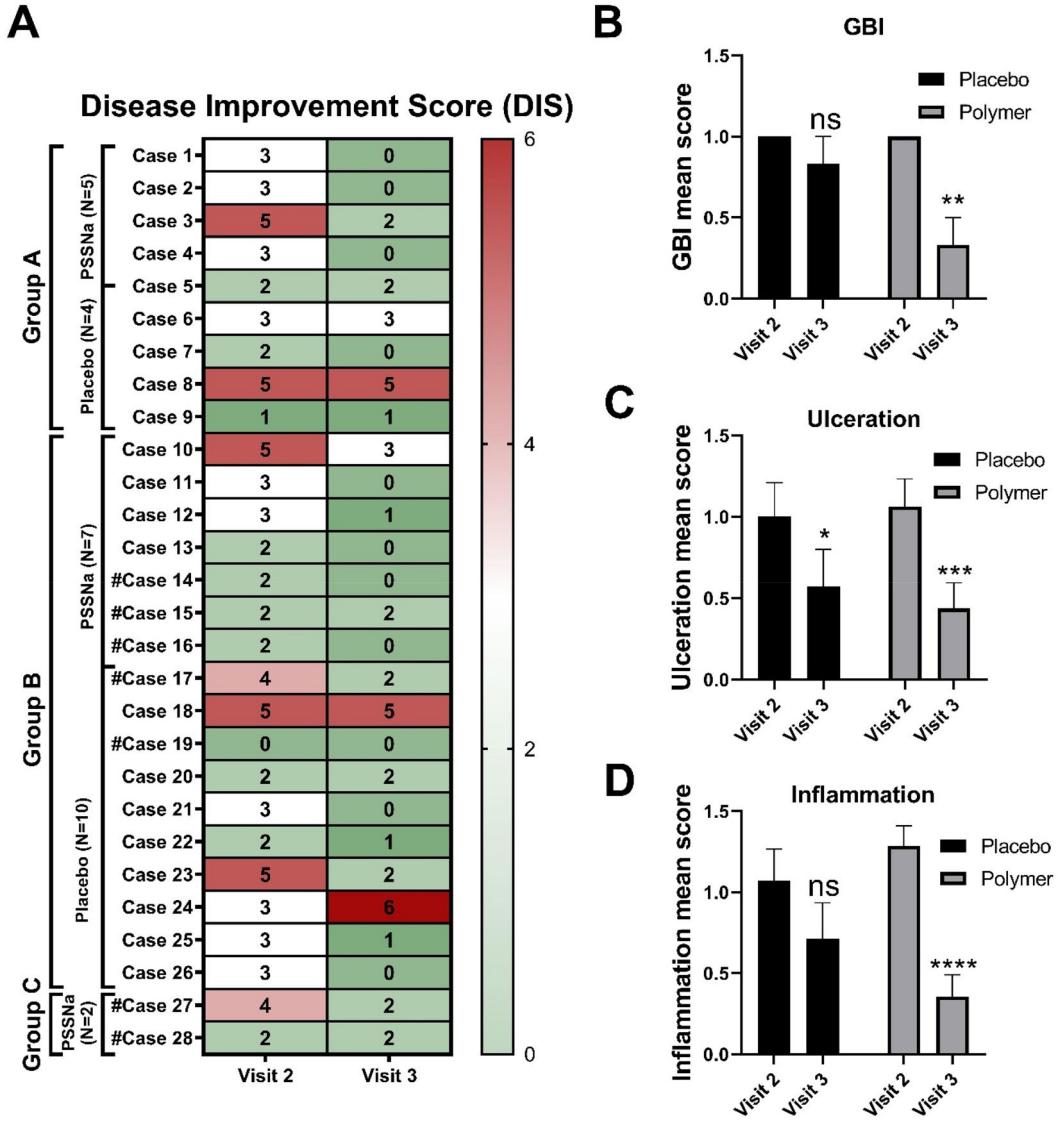
Changes in disease improvement scores (DIS). (A) DIS (GBI score [Gingival Bleeding Index] + ulceration score + inflammation score) between visit 1 (week 0) and visit 2 (week 2) or visit 3 (week 4) are presented for each case separately in the form of a heat-map for all groups (group A: juvenile gingivitis or older individuals; group B: gingivostomatitis, cats with teeth; group C: caudal stomatitis). The GBI improvement score was not included for cases 14, 15, 16, 17, 19, 27, and 28, as these cats had their teeth extracted. GBI (B), ulceration (C), and inflammation (D) mean improvement scores are presented for both placebo-treated (*N* = 14; *N* = 12 for GBI) and polymer-treated (*N* = 12; *N* = 9 for GBI) groups of cats. 2—small improvement/no improvement, 1—medium improvement, and 0—significant improvement/complete recovery. Data are presented as mean ± SEM. Differences between groups were compared using the mixed-effects model with the Geisser-Greenhouse correction and Dunnett’s multiple comparisons test. ns: not significant (*p* > 0.05), *p < 0.05, **p < 0.01, ***p < 0.001, ****p < 0.0001.

**Figure 6.**
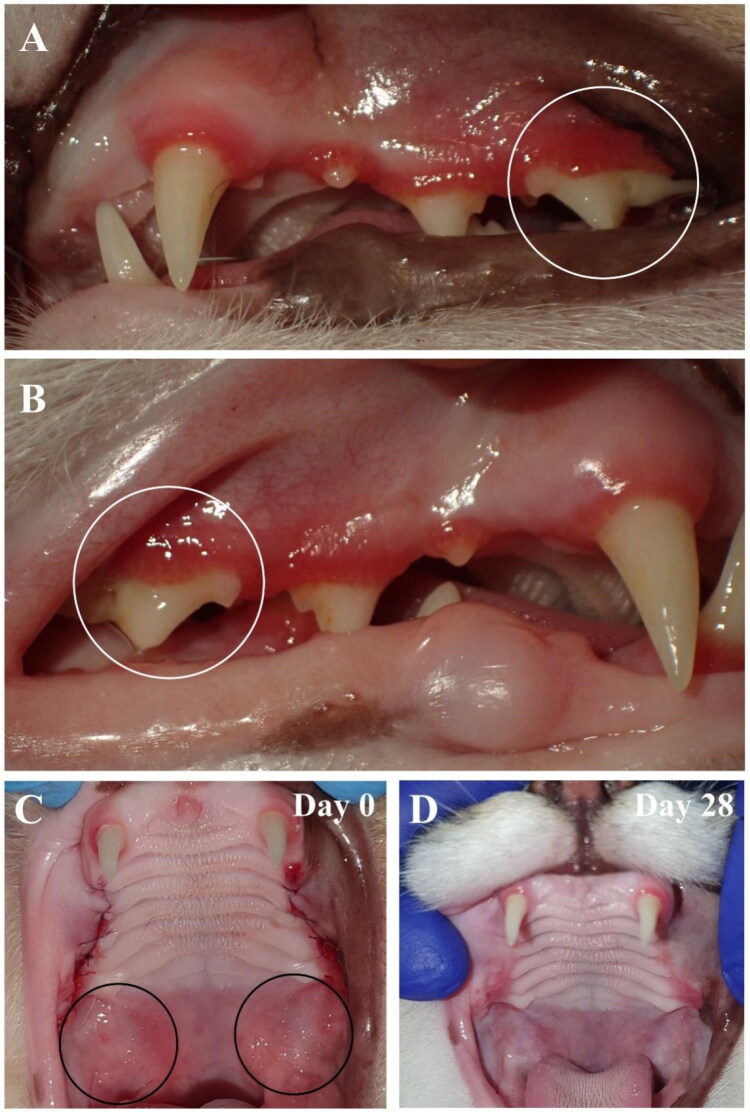
Cat FCV-positive receiving PSSNa (case 1, group A). (A,B) Preoperative assessment illustrating gingivitis stage 2 and caudal buccal mucosa inflamed with ulcerations (white circles); (C) postoperative picture after partial mouth extraction (PMX), which included all cheek dentition except canine teeth, visible caudal mucositis with ulcerations (black circles); (D) clinical control at day 28 with healed area previously inflamed.

**Figure 7.**
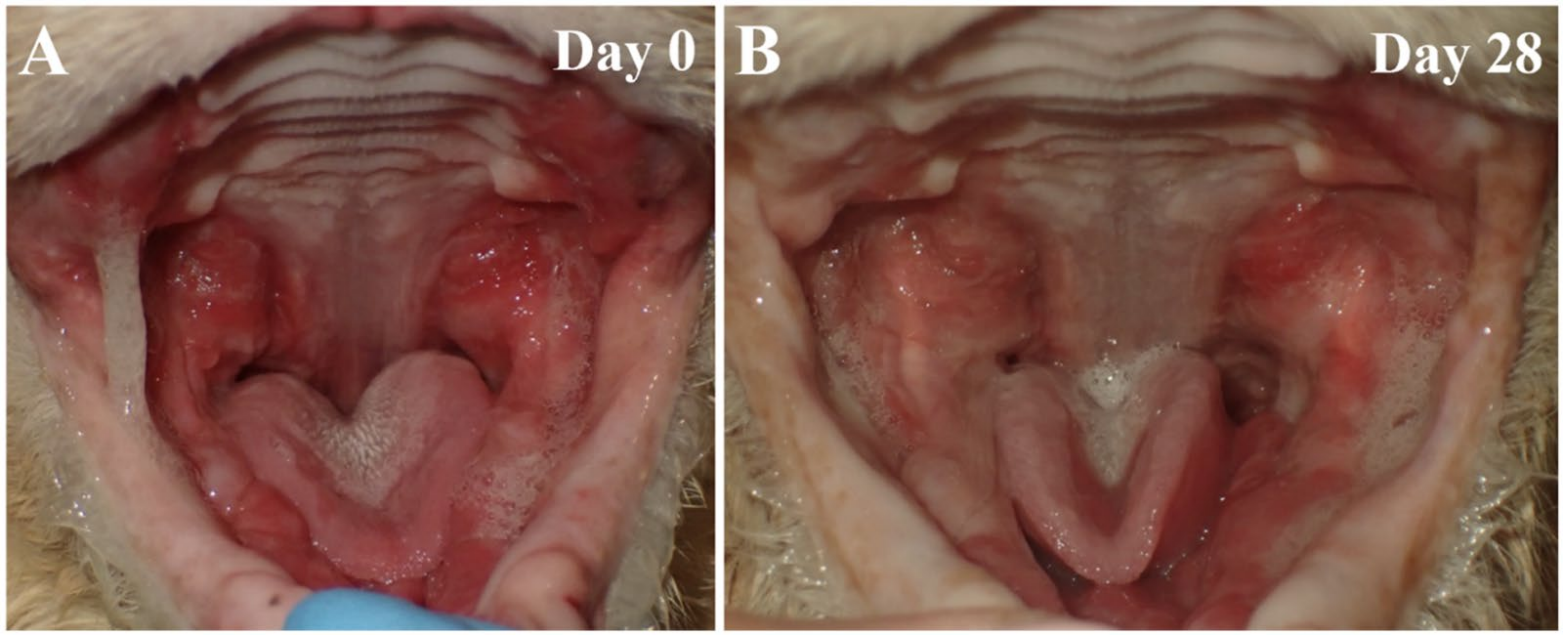
Cat Maine Coon FCV-positive receiving PSSNa, toothless at the moment of introduction (case 27, group C). (A) Cat with severe mucositis and ulcerations distributed symmetrically in the mouth on day 0 and (B) after 28 days of PSSNa daily application, resulting in reduced inflamed and ulcerated area.

The Disease Improvement Score (DIS) involves the Gingival Bleeding Index (GBI), the presence of ulceration, and the level of inflammation. DIS was assessed during visits 2 and 3, by comparing the cats’ conditions to those observed during visit 1. We found a marked improvement in all examined symptoms exclusively in the group treated with PSSNa. The placebo group showed some improvement during standard therapy, but it was not statistically significant. Notably, 7 out of 14 (50%) cats receiving PSSNa recovered completely from gingival bleeding, ulceration, and inflammation, compared to only 4 out of 14 (29%) cats in the placebo group which demonstrated similar recovery. The individual changes in OHI scores are presented in Figure 5(A). The changes in DIS scores are presented in Figures 5(B–D). The GBI score was not included for cases 14, 15, 16, 17, 19, 27, and 28, as cats had their teeth extracted. The representative pictures of the PSSNa-treated cats, case 1 and case 27, are presented in Figures 6 and 7, respectively. The general DIS change is noticeable. The first example is Case 1, a cat in Group A, which was assessed preoperatively and showed gingivitis stage 2, along with inflamed caudal buccal mucosa featuring ulcerations (see Figures 6(A,B), marked with white circles). A post-operative image was taken after a partial mouth extraction (PMX)—which included all cheek teeth except for the canine teeth—revealing visible caudal mucositis with ulcerations (see Figure 6(C), marked with black circles). After 28 days of treatment with PSSNa, the previously inflamed areas were completely healed (see Figure 6(D)). In another example, Case 27 was edentulous upon introduction and, therefore, placed in Group C. On Day 0, the cat displayed severe mucositis and ulcerations symmetrically throughout the mouth (see Figure 7(A)). Following 28 days of daily PSSNa application, there was a significant reduction in the inflammation and ulceration of the affected areas (see Figure 7(B)). The individual OHI and DIS clinical scores obtained throughout the study are presented in Supplementary Table 1.

Each follow-up visit included a general assessment of the patient’s condition. Both owners and veterinarians observed a significant improvement in cats treated either with a placebo or PSSNa. Slightly higher improvement was noted for the PSSNa-treated group, mainly when assessed by veterinarians (Table 3).

**Table 3.** Owner’s and clinical assessments of patients’ conditions.

	Owner’s assessment				Clinical assessment			
	Placebo		PSSNa		Placebo		PSSNa	
	Follow-up visit 1	Follow-up visit 2	Follow-up visit 1	Follow-up visit 2	Follow-up visit 1	Follow-up visit 2	Follow-up visit 1	Follow-up visit 2
Significant improvement	10/14 (71%)	10/14 (71%)	12/14 (86%)	10/14 (71%)	11/14 (79%)	10/14 (71%)	13/14 (93%)	12/14 (86%)
No improvement	4/14 (29%)	3/14 (21%)	3/14 (21%)	2/14 (14%)	3/14 (21%)	3/14 (21%)	1/14 (7%)	2/14 (14%)
Worsening of symptoms	0/14	1/14 (7%)	0/14	2/14 (14%)	0/14	1/14 (7%)	0/14	0/14

### Phylogenetic analysis reveals gene-specific clustering of PSSNa-resistant isolates

Nanopore sequencing enabled the acquisition of nucleotide sequences of genome fragments, specifically the ORF1 and ORF2 of FCV strains analyzed in this study. To evaluate potential regions of the viral genome that may be responsible for the PSSNa resistance, we focused on FCV strains isolated from cats treated with the polymer. We obtained complete sequences of the major capsid protein (VP1) gene, as well as the RNA-dependent RNA polymerase (RdRp) region. Case 6 was excluded from ORF2 phylogenetic analysis due to poor sequencing coverage and excessive ambiguous nucleotides in this region. Phylogenetic reconstruction based on translated amino acid sequences of the RdRp and VP1 regions, combined with reference sequences of FCV genomes available in the GenBank database, revealed distinct clustering patterns for ORF1 and ORF2 regions with varying degrees of resistance-associated grouping.

### ORF1 (RdRp) phylogenetic analysis

The dataset for ORF1 analysis comprised 11 samples: 3 drug-resistant isolates (cases 5, 6, and 28), marked in red; 7 drug-susceptible isolates (cases 1, 2, 11, 12, 14, 16, and 27), marked in blue; and the reference strain F9 marked in grey (see Supplementary Figure 5). Maximum likelihood analysis of ORF1 sequences demonstrated clear clustering of drug-resistant isolates within a monophyletic clade supported by strong statistical evidence (SH-aLRT support: 100/100, ultrafast bootstrap: 100%). The cases 5, 6, and 28 originated from cats that were resistant to treatment cluster together on the phylogenetic tree (see Supplementary Figure 5, marked in red), which indicates that they are closely related and suggests that the RdRp protein sequence may be associated with lack of response to PSSNa treatment in cats infected with FCV.

The branch lengths within this resistant cluster ranged from 0.0234 to 0.0564 substitutions per site, indicating moderate sequence divergence despite shared resistance phenotype. Notably, drug-susceptible case 11 (case 11: 0.0504 substitutions per site) clustered within the resistant clade with high confidence (SH-aLRT: 90.5/100), suggesting phylogenetic proximity does not strictly correlate with resistance phenotype. This isolate may represent an intermediate evolutionary state or harbor cryptic resistance mutations not expressed under current selective conditions. The resistant clade was positioned as a sister group to other major FCV lineages with moderate support (SH-aLRT: 67.4/84, bootstrap: 62%), indicating these variants share a common evolutionary origin.

### ORF2 (VP1) phylogenetic analysis

The dataset for ORF1 analysis comprised 11 samples: 2 drug-resistant isolates (cases 5 and 28), marked in red; 8 drug-susceptible isolates (cases 1, 4, 10, 11, 12, 14, 16, and 27), marked in blue; and the reference strain F9 marked in grey (see Supplementary Figure 6). ORF2 phylogenetic analysis revealed partial clustering of drug-resistant isolates. While not forming a complete monophyletic group as observed in ORF1, drug-resistant isolates showed evidence of shared evolutionary relationships: (a) Case 5: Clustered with case 4 (susceptible) in a well-supported clade (95.4/82 support) with branch length 0.1741; (b) Case 6: Excluded from ORF2 analysis due to poor sequencing coverage and excessive ambiguous nucleotides; (c) Case 28: Formed a supported clade with case 11 (susceptible) with moderate to strong support (81.1/85), suggesting potential VP1-mediated resistance mechanisms in a subset of strains and indicating that case 11 may harbor resistance-associated genetic changes in both ORF1 and ORF2 regions.

The ORF2 tree topology showed extensive polytomies and shorter branch lengths overall, consistent with conservation of essential structural protein functions, yet revealed resistance-associated groupings distinct from the random distribution initially expected.

### Statistical support interpretation

Branch support values in both trees represent dual statistical measures: SH-aLRT support percentages (first value) and ultrafast bootstrap percentages (second value). Values ≥95% indicate strong support, 80-94% moderate support, and <80% weak support. Branch lengths represent evolutionary distance measured in amino acid substitutions per site, with longer branches indicating greater sequence divergence.

## Discussion

The results of this preliminary study provide substantial evidence supporting the safety and efficacy of poly(sodium 4-styrenesulfonate) (PSSNa) as a candidate for antiviral treatment for feline calicivirus (FCV) infections. Previously, we demonstrated that PSSNa effectively inhibited FCV and FHV-1 replication *in vitro* through distinct mechanisms: PSSNa blocks FHV-1 by preventing virion entry into host cells and inhibits FCV replication by interfering with its replication cycle (Synowiec et al. 2019). This study extends these findings by evaluating the safety profile and therapeutic potential of PSSNa *in vivo*, specifically in cats suffering from FCV-related oral inflammatory diseases. Currently, treatment options for FCV are limited, especially for cats with chronic conditions, kittens, or those in environments where consistent antiviral therapy is difficult to administer. Existing prevention strategies primarily rely on vaccination, which may not be universally effective due to the genetic diversity of FCV strains. Furthermore, there is a lack of direct antiviral treatments specifically targeting FCV, highlighting the unmet need for supportive therapies like that involving PSSNa administration. In this study, PSSNa demonstrated a significant reduction in viral load and clinical symptoms, presenting an approach that could serve a diverse feline population, including those with chronic oral diseases or comorbidities. The ease of administration and lack of toxicity make PSSNa a candidate for further evaluation in adjunctive therapy or supplemental treatment.

The study cohort included cats with varying stages of oral inflammatory diseases, adding to the robustness of our findings. Except for Case 5, every PSSNa-treated cat exhibited clinical improvement, whereas five cats in the placebo-treated group did not show any clinical improvement. This supports the conclusion that PSSNa benefits affected cats as a supportive therapy. Notably, cases 2, 3, 4, 11, 13, 16, and 27, treated with PSSNa, were FCV-free at the end of the study, and they showed noticeable clinical improvement. These cases underscore the potential of PSSNa to achieve both virological and clinical success. However, some placebo-treated cats (Cases 7, 21, and 23) also became FCV-free, indicating that standard therapy can still be effective in some cases and demonstrating that existing treatments can sometimes suffice to combat the virus. This observation aligns with previous studies showing that the extraction of teeth in areas of oral inflammation provides a substantial improvement or complete resolution of stomatitis (Jennings et al. 2015). Additionally, placebo-treated cases 19, 20, 25, and 26 showed no change or increase (case 22) in FCV load but exhibited clinical improvement, suggesting that FCV is not always the leading cause of gingivitis. In contrast, in placebo-treated cases such as 6, 8, and 18, no or slight changes in FCV load were associated with no clinical improvement, emphasizing the multifactorial nature of oral inflammatory diseases. The authors did not observe the influence of age, sex, or body weight on the obtained results. None of the cats subjected to the study suffered from metabolic conditions, such as chronic kidney disease, diabetes, or cardiopulmonary disorders. Nutrition was advised based on the treatment profile and was soft, moistened, and highly digestible.

Having in mind previous reports suggesting no direct association between viral load and clinical outcomes (e.g. Druet and Hennet 2017), we have also shown an improvement in clinical symptoms during PSSNa treatment, which corresponded with a reduction in viral load. While this discrepancy may be attributed to the treatment itself, it could also be related to the genetic variability of the FCV strains. To verify this hypothesis, we performed genotyping of the isolates, focusing on the FCV strains from PSSNa-treated cats. The analysis revealed that the isolates exhibit exceptionally high genetic diversity, underlining the efficacy of PSSNa against diverse strains. Second, the phylogenetic analysis of the ORF1 and ORF2 suggests a correlation between the resistance to treatment and genetic identity. However, due to the high variability of the isolates, we are not able to unequivocally identify individual amino acids or motifs responsible for the resistance, nor can we exclude that certain strains are more aggressive, hence the lack of improvement. This may be even more complex, as our *in vitro* studies showed that PSSNa inhibits FCV at both entry and replication steps (Synowiec et al. 2019). Interestingly, our results also show that the F9 reference (vaccine) strain clusters far from the currently circulating FCV strains in the feline population, which is in contrast to the previous studies (Smith et al. 2020).

Several limitations of this study should be acknowledged. First, it was the pet owners and not veterinarians who administered the compound, introducing variability in the consistency and accuracy of the administration protocol. Differences in owner technique and adherence to the instructions may have influenced the observed outcomes and could contribute to variability in the data. Nonetheless, this design closely mirrors the intended real-world use of the PSSNa, providing valuable insight into its practicality and feasibility under typical field conditions. Second, the cats included in the study were not housed in sterile conditions, and each case represented different microbial and physical statuses, which could influence outcomes. However, this approach also reflects real-world conditions in which the treatment is intended to be used, providing valuable information on its performance under typical field circumstances. Additionally, the diversity of oral disease severity and concurrent treatments among the cats adds complexity to interpreting the results. Finally, the modest sample size limits the strength of statistical conclusions. Taken together, while these limitations warrant cautious interpretation, the study offers a valuable first exploration of the PSSNa’s field efficacy and sets the stage for the larger, more controlled investigations. Finally, while clinical improvements were observed, the subjective nature of some assessment metrics could introduce bias, underscoring the need for more objective and quantitative evaluation tools.

Our study highlights the importance of adjuvant therapy in managing FCV-related diseases. While PSSNa does not revolutionize treatment approaches, which remain focused on dental and surgical interventions, it provides a valuable complementary tool for managing viral load and alleviating clinical symptoms. Interestingly, while no improvement in the Oral Health Index (OHI) was observed in some cases, a significant change in the Disease Improvement Score (DIS) was noted. This suggests that DIS may be more closely connected to FCV-related symptoms and could be a more relevant metric for assessing clinical outcomes in FCV-associated stomatitis. These findings should be interpreted cautiously, given the exploratory nature of the study and the modest sample size. Nonetheless, the observed changes in DIS provide preliminary evidence that warrants further investigation in larger, controlled studies to fully assess the potential benefits of the treatment.

In conclusion, PSSNa demonstrated antiviral activity, is non-toxic, and can be easily administered, suggesting it may warrant further investigation for use in cats suffering from FCV infections. Its favorable safety profile and its significant efficacy in reducing viral load and improving clinical outcomes make it an ideal candidate for adjunct therapy. The ease of administration also facilitates application by pet owners, broadening its applicability as supplementary therapy for managing FCV-related conditions. Future studies should address the limitations of this study, including the characterization of FCV strains and the refinement of clinical assessment tools, to further establish PSSNa as a reliable antiviral option for the feline population.

## Supplementary Material

Supplemental Material

Supplementary Table Patients course.docx

Supplementary Material 3 Skin irritation.xlsx

Supplementary Material 1 Microbiological Purity Test Result.xlsx

Supplementary Material 2 Aging and packaging compliance test.xlsx
