# Supplementary material for: Antiviral effect of poly(styrene 4-sulfonate) (PSSNa) on feline calicivirus oral infections in cats—field study
Source: Vet Q. 2026 Jan 19;46(1):2616395. doi: 10.1080/01652176.2026.2616395 (PMC12818315; doi:10.1080/01652176.2026.2616395)
Supplement: Supplemental Material [file TVEQ_A_2616395_SM2290.docx]

Supplementary Figure 1.


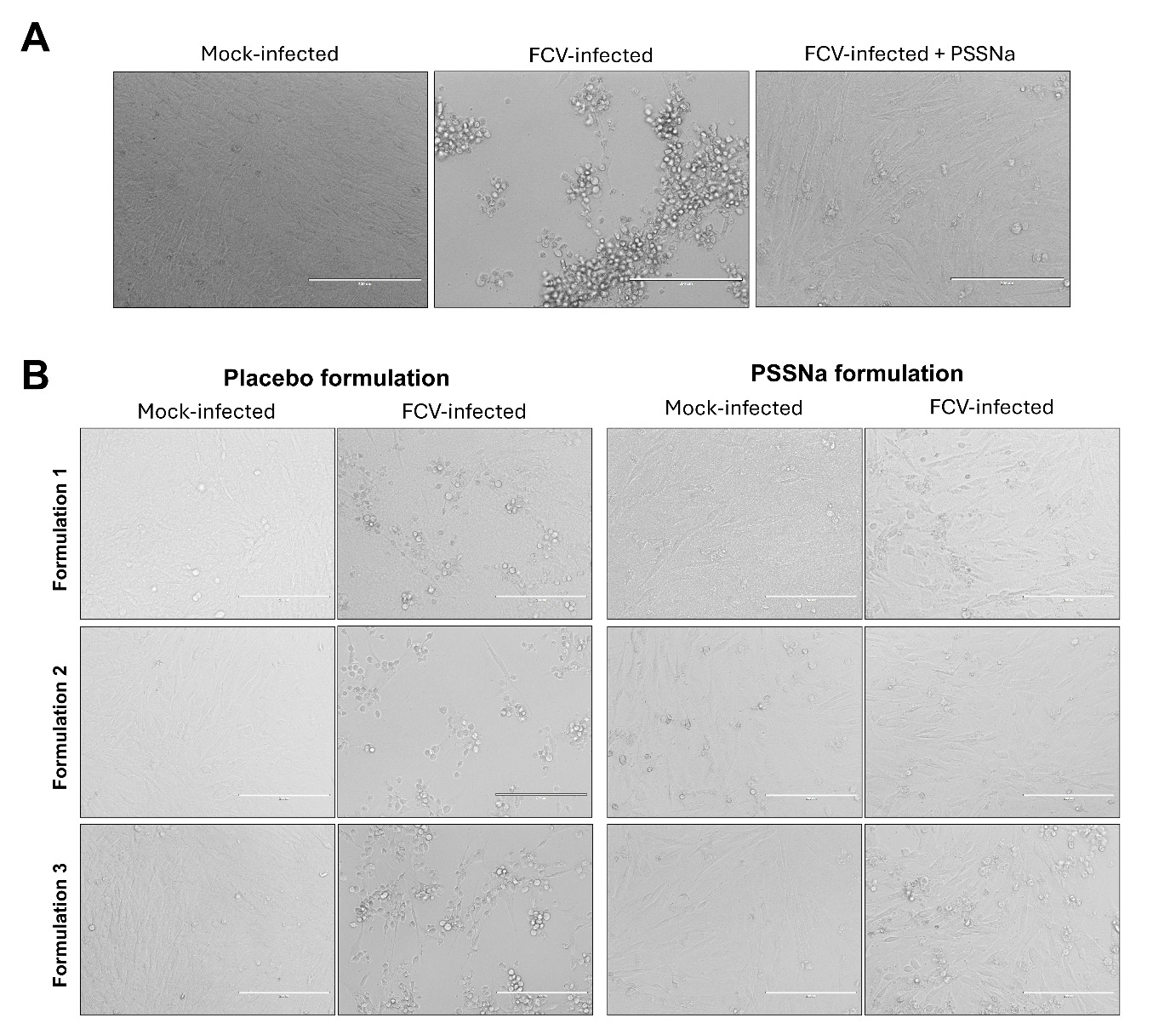


**Supplementary Figure 1. Tested formulations retain anti-FCV activity.** Experiments were carried out at a concentration of 200 μg/ml. Assessment of FCV-induced CPE was observed after 24 h post-infection for (**A**) control inoculum (DMEM medium) and for (**B**) formulations. The scale bar denotes 200 µm.

Supplementary Figure 2.


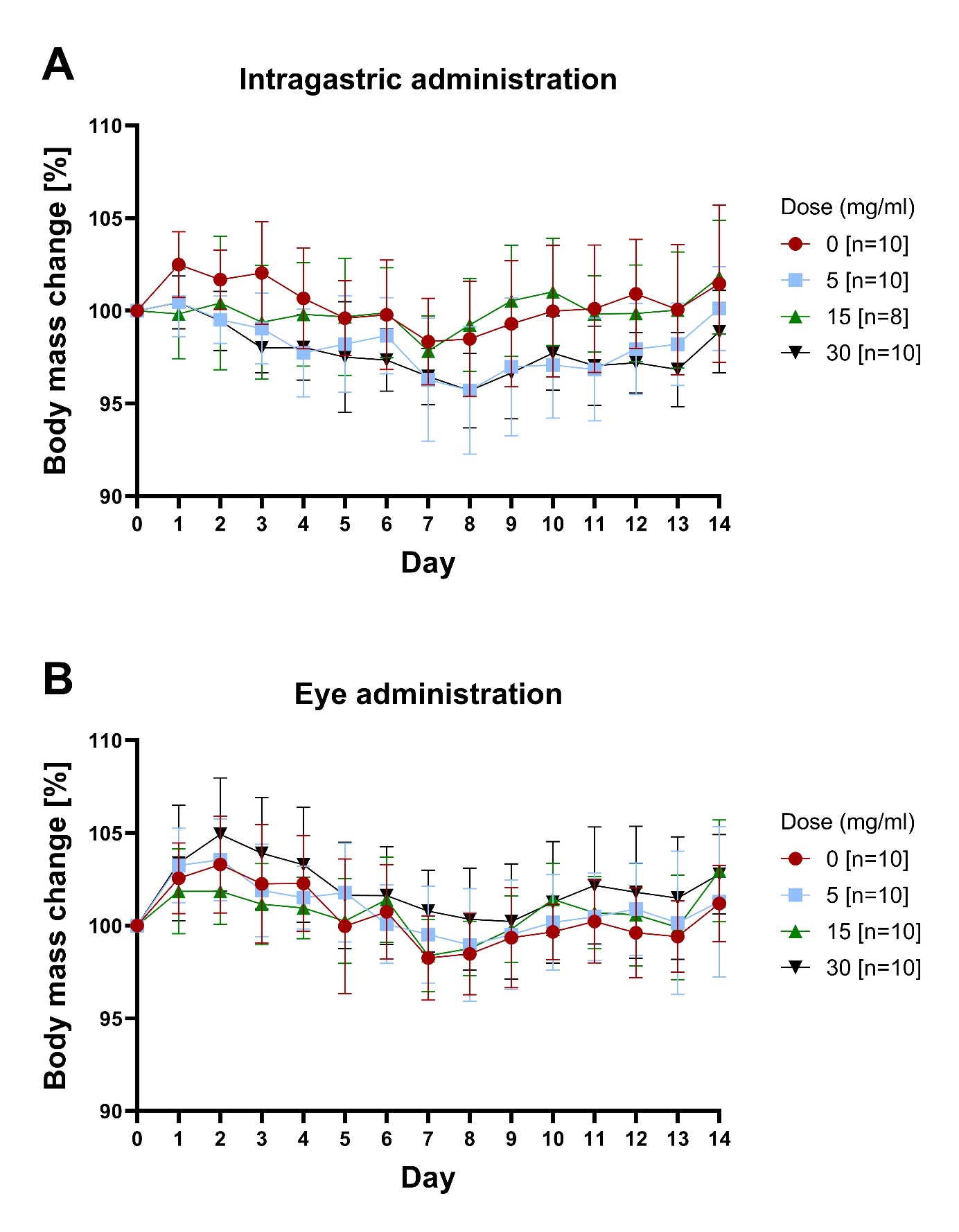


**Supplementary Figure 2. Changes in body weight in mice after administration of PSSNa.** Mice were monitored and weighed daily. After 14 days from the first application, animals were euthanized by cervical dislocation. The study was carried out in two independent repeats (5 animals per repeat). Data are presented as mean ± SEM.

Supplementary Figure 3.


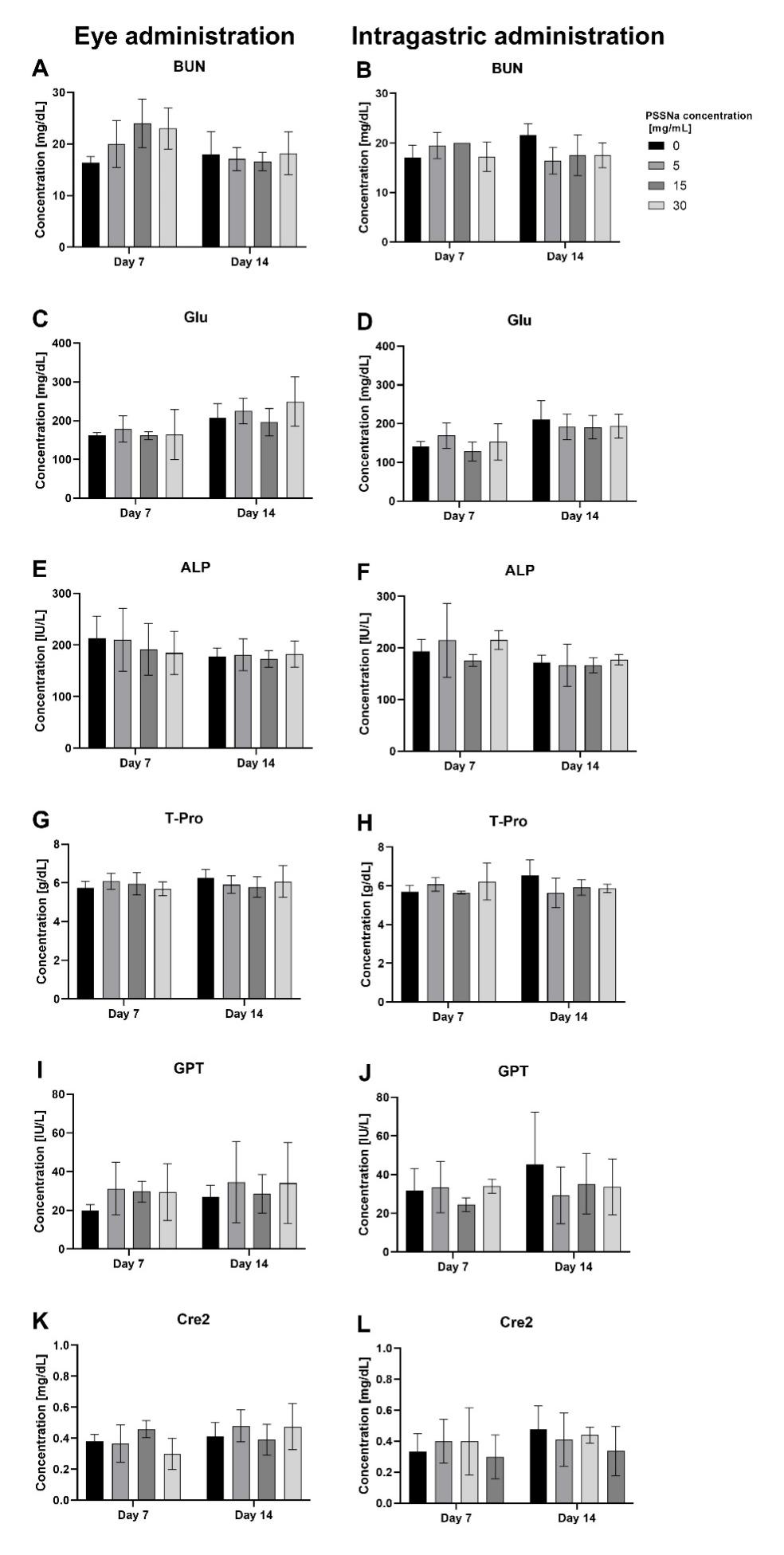
**Supplementary Figure 3. PSSNa treatment does not change blood parameters.** Mice were monitored and weighed daily. After 14 days from the first application, animals were euthanized by cervical dislocation. The blood, liver, kidney, and spleen were collected from euthanized mice for further analysis. The biochemical analysis, including GLU, BUN, ALP, TP, GPT, and CRE, was performed on days 7 and 14. The study was carried out in two independent repeats (5 animals per repeat). Data are presented as mean ± SEM.

Supplementary Figure 4.


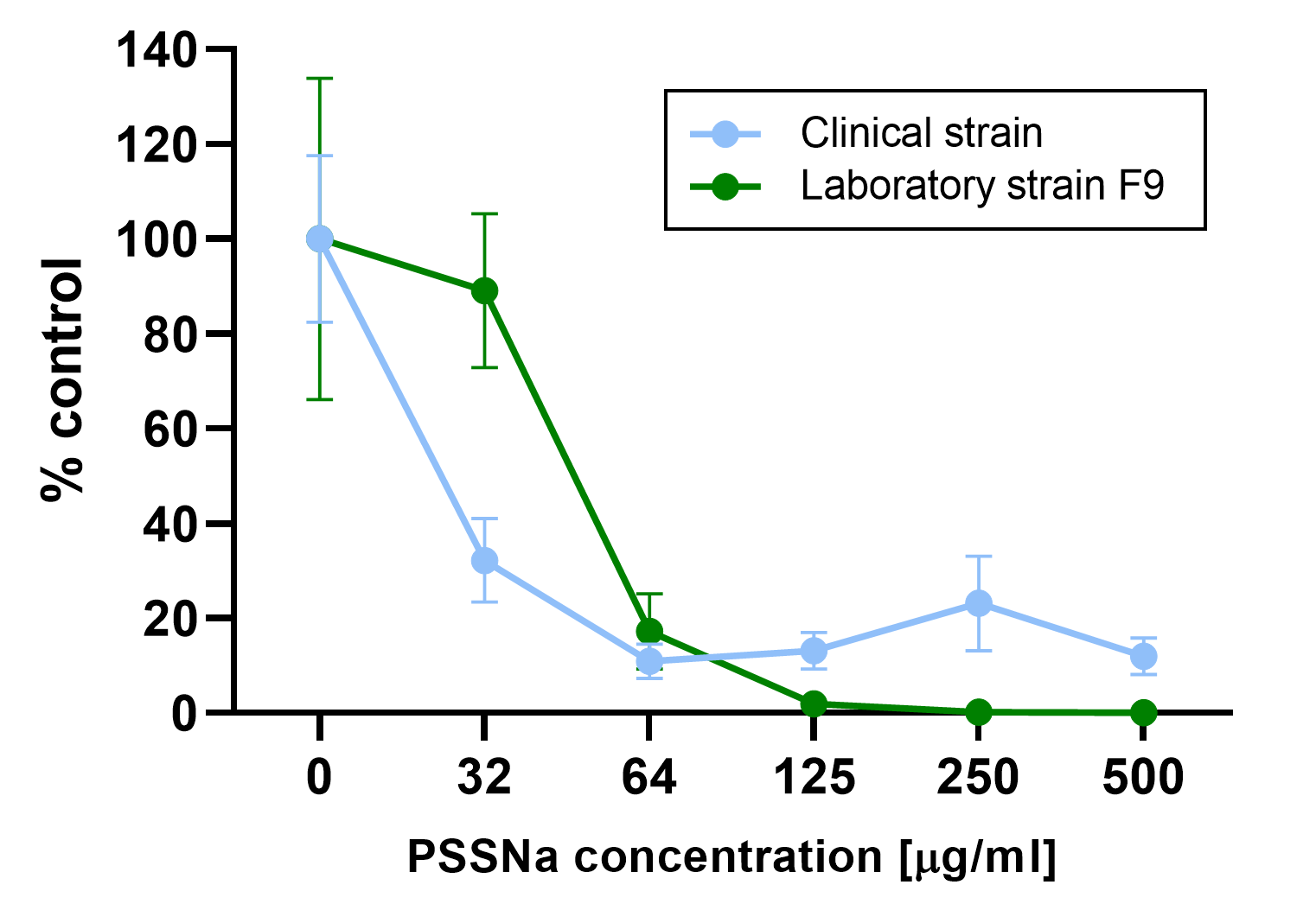


**Supplementary Figure 4. PSSNa effectiveness comparison between FCV laboratory strain F9 and FCV clinical strain isolated from the cat with resistant FCV infection (case 28).** Cells were infected with FCV clinical strain or reference laboratory strain F9. Experiments were carried out at different concentrations of PSSNa: 0, 32, 64, 125, 250, or 500 µg/ml. Virus yield was assessed by RT-qPCR, and the data is presented as a % control of the number of virus RNA copies/ml. Results were normalized against the values obtained for untreated, infected cells and are presented as mean ± SEM. The data presented was collected in three independent experiments, each performed at least in duplicate.

Supplementary Figure 5.


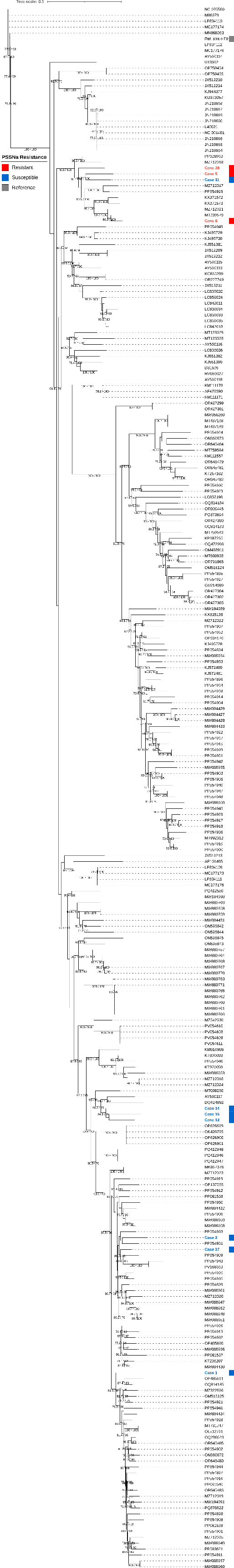

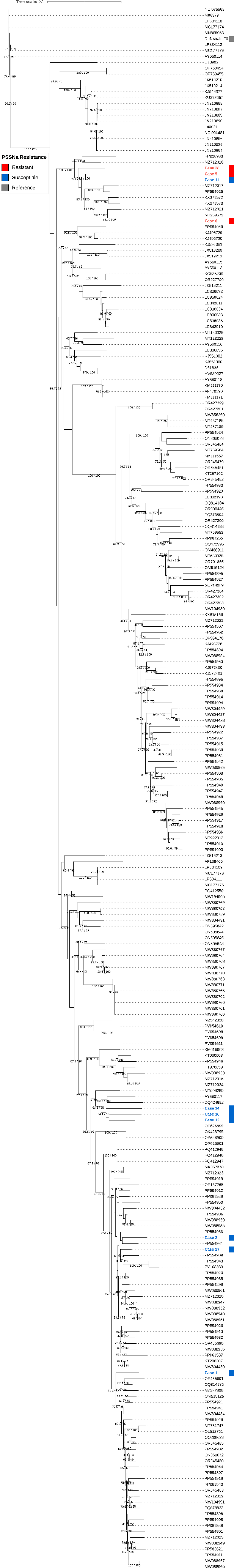


**Supplementary Figure 5. Maximum likelihood phylogenetic tree of FCV ORF1 (RdRp) sequences.** Drug-resistant isolates are marked in red, and drug-susceptible isolates are marked in blue. Branch support values represent SH-aLRT support/ultrafast bootstrap percentages. Branch lengths are proportional to evolutionary distance (substitutions per site). Drug-resistant samples form a well-supported monophyletic clade, indicating a common evolutionary origin of resistance mechanisms.

Supplementary Figure 6.


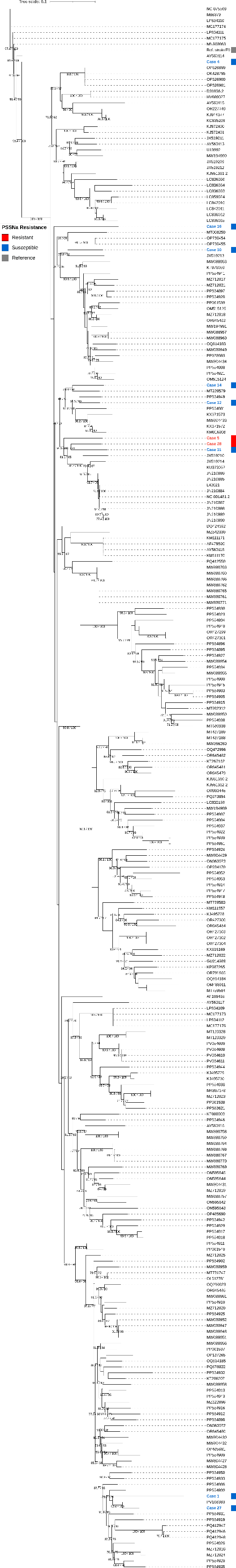

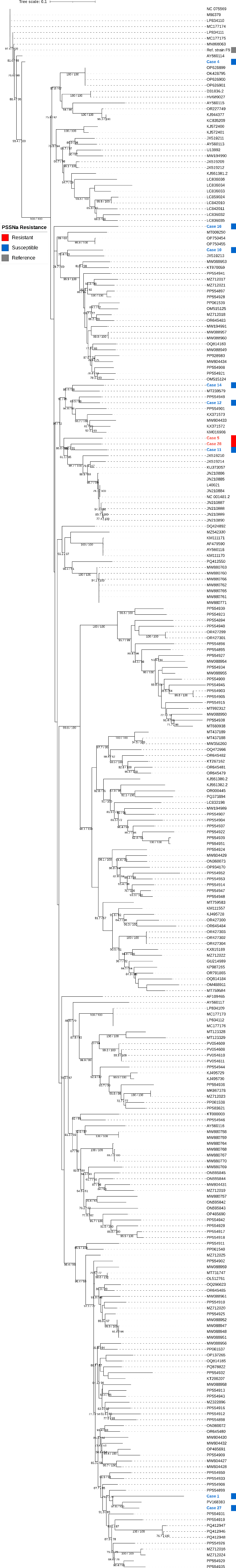


**Supplementary Figure 6. Maximum likelihood phylogenetic tree of FCV ORF2 (VP1) sequences**. Drug-resistant isolates are marked in red, and drug-susceptible isolates are marked in blue. Branch support values represent SH-aBranch support values represent SH-aLRT support/ultrafast bootstrap percentages. Branch lengths are proportional to evolutionary distance (substitutions per site). Drug-resistant cases show partial clustering patterns, with some cases forming supported clades while others distribute among susceptible isolates. Case 6 was excluded due to poor sequencing coverage in the ORF2 region.
