# Supplementary material for: Antiviral effect of poly(styrene 4-sulfonate) (PSSNa) on feline calicivirus oral infections in cats—field study
Source: Vet Q. 2026 Jan 19;46(1):2616395. doi: 10.1080/01652176.2026.2616395 (PMC12818315; doi:10.1080/01652176.2026.2616395)
Supplement: Supplementary Table Patients course.docx [file TVEQ_A_2616395_SM9405.docx]

**Supplementary Table 1. Patient’s clinical course for: Group A (juvenile gingivitis or older individuals), Group B (gingivostomatitis – cats with teeth), Group C (caudal stomatitis – toothless cat)**

|  | **Date of qualification** | **Treatment** | **Initial control** | | | **2 weeks control** | | | | | | **Final control** | | | | | | |
| --- | --- | --- | --- | --- | --- | --- | --- | --- | --- | --- | --- | --- | --- | --- | --- | --- | --- | --- |
|  |  |  | **LN** | **DD** | **P** | **LN** | **DD** | **P** | **GBI** | **U** | **I** | **LN** | **DD** | **P** | **GBI** | **U** | **I** | **Viral load change*** |
| **GROUP A** | | | | | | | | | | | | | | | | | | |
| Case 1 | 4/2022 | PSSNa | 1 | 1 | 1 | 1 | 0 | 1 | 1 | 1 | 1 | 0 | 0 | 0 | 0 | 0 | 0 | Decrease |
| Case 2 | 5/2022 | PSSNa | 2 | 1 | 1 | 1 | 0 | 1 | 1 | 1 | 1 | 1 | 0 | 0 | 0 | 0 | 0 | Decrease (complete recovery) |
| Case 3 | 5/2022 | PSSNa | 1 | 1 | 1 | 1 | 1 | 1 | 1 | 2 | 2 | 1 | 1 | 0 | 0 | 2 | 0 | Decrease (complete recovery) |
| Case 4 | 12/2022 | PSSNa | 1 | 1 | 1 | 1 | 1 | 1 | 1 | X | 2 | 1 | 0 | 0 | 0 | 0 | 0 | Decrease (complete recovery) |
| Case 5 | 2/2023 | PSSNa | 2 | 1 | 1 | 1 | 1 | 1 | 1 | 0 | 1 | 2 | 1 | 1 | 1 | 0 | 1 | Slight increase |
| Case 6 | 10/2022 | Placebo | 1 | 1 | 0 | 0 | 1 | 1 | 1 | 1 | 1 | 0 | 1 | 1 | 1 | 1 | 1 | No change |
| Case 7 | 1/2023 | Placebo | 1 | 1 | 0 | 0 | 0 | 1 | 1 | 0 | 1 | 0 | 0 | 0 | 0 | 0 | 0 | Decrease (complete recovery) |
| Case 8 | 1/2023 | Placebo | 1 | 1 | 0 | 0 | 1 | 1 | 1 | 2 | 2 | 0 | 1 | 1 | 1 | 2 | 2 | Slight decrease |
| Case 9 | 2/2023 | Placebo | 0 | 1 | 1 | 0 | 0 | 1 | 1 | 0 | 0 | 0 | 0 | 1 | 1 | 0 | 0 | Slight decrease |
| **GROUP B** | | | | | | | | | | | | | | | | | | |
| Case 10 | 3/2022 | PSSNa | 1 | 1 | 2 | 1 | 0 | 1 | 1 | 2 | 2 | 1 | 1 | 1 | 1 | 1 | 1 | Decrease |
| Case 11 | 4/2022 | PSSNa | 1 | 2 | 2 | 0 | 0 | 1 | 1 | 1 | 1 | 0 | 0 | 0 | 0 | 0 | 0 | Decrease (complete recovery) |
| Case 12 | 7/2022 | PSSNa | 2 | 1 | 2 | 2 | 2 | 2 | 1 | 1 | 1 | 1 | 0 | 1 | 1 | 0 | 0 | Slight decrease |
| Case 13 | 10/2022 | PSSNa | 1 | 1 | 1 | 1 | 0 | 1 | 1 | 0 | 1 | 1 | 0 | 0 | 0 | 0 | 0 | Decrease (complete recovery) |
| Case 14 | 12/2022 | PSSNa | 2 | 2 | 2 | 1 | NA | 1 | NA | 1 | 1 | 1 | NA | NA | NA | 0 | 0 | Decrease |
| Case 15 | 1/2023 | PSSNa | 2 | 2 | 2 | 2 | NA | 1 | NA | 1 | 1 | 1 | NA | NA | NA | 1 | 1 | Data missing |
| Case 16 | 1/2023 | PSSNa | 2 | 2 | 2 | 1 | NA | 1 | NA | 1 | 1 | 0 | NA | NA | NA | 0 | 0 | Decrease (complete recovery) |
| Case 17 | 4/2022 | Placebo | 2 | 2 | 2 | 2 | NA | NA | NA | 2 | 2 | 1 | NA | NA | NA | 1 | 1 | Decrease |
| Case 18 | 4/2022 | Placebo | 1 | 1 | 2 | 1 | 1 | 2 | 1 | 2 | 2 | 1 | 1 | 2 | 1 | 2 | 2 | Slight decrease |
| Case 19 | 6/2022 | Placebo | 2 | 2 | 2 | 1 | NA | NA | NA | 0 | 0 | 0 | NA | NA | NA | 0 | 0 | No change |
| Case 20 | 6/2022 | Placebo | 1 | 2 | 2 | 1 | 1 | 1 | 1 | 0 | 1 | 1 | 1 | 1 | 1 | 0 | 1 | Slight decrease |
| Case 21 | 9/2022 | Placebo | 1 | 1 | 2 | 1 | 0 | 1 | 1 | 1 | 1 | 0 | 0 | 0 | 0 | 0 | 0 | Decrease (complete recovery) |
| Case 22 | 10/2022 | Placebo | 1 | 2 | 2 | 1 | 1 | 1 | 1 | 1 | 0 | 0 | 1 | 1 | 1 | 0 | 0 | Slight increase |
| Case 23 | 11/2022 | Placebo | 1 | 2 | 2 | 1 | 1 | 1 | 1 | 2 | 2 | 0 | 0 | 1 | 1 | 0 | 1 | Decrease (complete recovery) |
| Case 24 | 1/2023 | Placebo | 1 | 2 | 2 | 1 | 1 | 1 | 1 | 1 | 1 | 1 | 1 | 2 | 2 | 2 | 2 | Decrease |
| Case 25 | 1/2023 | Placebo | 2 | 1 | 1 | 0 | 0 | 1 | 1 | 1 | 1 | 0 | 0 | 1 | 1 | 0 | 0 | No change |
| Case 26 | 2/2023 | Placebo | 1 | 2 | 2 | 1 | 0 | 1 | 1 | 1 | 1 | 0 | 0 | 0 | 0 | 0 | 0 | No change |
| **GROUP C** | | | | | | | | | | | | | | | | | | |
| Case 27 | 2/2022 | PSSNa | 2 | NA | NA | 2 | NA | NA | NA | 2 | 2 | 2 | NA | NA | NA | 1 | 1 | Decrease (complete recovery) |
| Case 28 | 4/2022 | PSSNa | 2 | 1 | 1 | 1 | NA | NA | NA | 1 | 1 | 1 | NA | NA | NA | 1 | 1 | No change |

LN – lymph nodes; DD – dental deposits; P – periodontium; GBI – gingival bleeding; U – ulceration; I – inflammation; NA – non-applicable *Decrease (>10x), slight decrease (2-10x), no change, slight increase (2-10x), increase (>10x)
